# Microstructure and Corrosion Behavior of Friction Stir-Welded 6061 Al/AZ31 Mg Joints with a Zr Interlayer

**DOI:** 10.3390/ma12071115

**Published:** 2019-04-03

**Authors:** Yang Zheng, Xiaomeng Pan, Yinglei Ma, Shuming Liu, Libin Zang, Yong Chen

**Affiliations:** 1 School of Mechanical Engineering, Hebei University of Technology, Tianjin 300130, China; 201821202041@stu.hebut.edu.cn (Y.M.); 201611201012@stu.hebut.edu.cn (L.Z.); 2 Patent Examination Cooperation (Tianjin) Center of the Patent Office, CNIPA, Tianjin 300304, China; pan_xi_aomeng@126.com; 3 School of Chemical Engineering and Technology, Hebei University of Technology, Tianjin 300130, China; lsm9707@163.com

**Keywords:** friction stir welding, Al/Mg dissimilar joints, Zr interlayer, corrosion, microstructure

## Abstract

Friction stir welding (FSW) with a Zr interlayer was employed to join dissimilar alloys of 6061 Al and AZ31 Mg. The microstructures of Al/Mg and Al/Zr/Mg joints were investigated by optical microscopy (OM), scanning electron microscopy (SEM), and energy dispersive X-ray spectrometer (EDS). The results showed that the central part of the Zr interlayer was smashed and intermixed with the base materials in the stir zone, whereas the undamaged part remained stable at the Al/Mg interface. The formation of Al–Mg intermetallic compounds (IMCs) was suppressed by the Zr interlayer due to its synergetic effects of chemical modification and thermal barrier. The electrochemical measurements revealed a differentiated corrosion behavior for each joint, where the corrosion rate of representative regions increased in the order of Al alloy < Mg alloy < heat-affected zone < stir zone. The immersion tests indicated an enhancement in corrosion resistance for the Al/Zr/Mg joint compared with the Al/Mg joint, which is owing to the mitigated galvanic corrosion between the base materials by the Zr interlayer.

## 1. Introduction

Vehicle weight reduction is attracting unprecedented attention under increasingly strict emission regulations in recent years. Al and Mg alloys have been extensively studied to substitute steel parts to realize an obvious lightweight effect. The development of suitable welding techniques for these two alloys is vital and has become a hot research topic [1,2]. However, the undesirable Al–Mg intermetallic compounds (IMCs) with hard and brittle properties formed by the conventional fusion welding techniques seriously deteriorate the joint quality, which is the main obstacle for the dissimilar joining of Al and Mg alloys [3,4]. 

Friction stir welding (FSW), a solid-state welding technique developed by the Welding Institute (Cambridge, UK) in 1991, exhibits great potential to join Al and Mg dissimilar alloys [5,6,7,8]. Rao et al. [5] demonstrated the feasibility of FSW in joining 6022 Al and AM60B Mg alloys. A sound Al/Mg joint with a high failure load was obtained by FSW via building a favorable weld geometry and dispersed Al–Mg IMCs in the stir zone. The studies of Sato et al. [7] and Kostka et al. [8] showed that the formation of Al–Mg IMCs, such as Al_3_Mg_2_ and Al_12_Mg_17_ phases, can hardly be avoided owing to the constitutional liquation phenomenon. 

Various kinds of interlayers (including adhesive, pure metal, alloy, and oxide) have been introduced into Al/Mg FSW joints to improve their mechanical properties via reducing the Al–Mg IMCs [9,10,11,12]. For example, Chowdhury et al. [9] added a Terokal 5089 interlayer into the 5754 Al/AZ31B Mg FSW joint and found a significant enhancement in the lap shear strength and fatigue properties due to the lower amount of Al–Mg IMCs. Xu et al. [10] increased the failure load of a 2024 Al/AZ31 Mg FSW joint by depositing a hot-dipped Zn interlayer, which was ascribed to the beneficial microstructure of a brazed zone at the shoulder edge (composed of Mg–Zn and Al–Zn) and a transition zone in the hook region (constituted with MgZn_2_, Zn-rich phase, and residual Zn) instead of the Al–Mg IMCs. Gao et al. [12] reported that the plasma electrolytic oxidation (PEO) interlayer could suppress the formation of Al–Mg IMCs and increase the fracture load of an ADC12 Al/AMX602 Mg FSW joint by mitigating the Al/Mg interfacial reaction under proper welding parameters. 

The research emphasis at present relating to Al/Mg FSW joints with interlayers is focused on the enhancement of the mechanical properties. In addition to the mechanical properties, corrosion properties are another key issue for joint safety in the service course [13,14]. In general, preferential corrosion occurs at the Mg alloy in Al/Mg FSW joints because of its lower corrosion potential, which will damage the joint strength and lead to joint failure, compared with that of the Al alloy [15,16]. Therefore, it is necessary to regulate the corrosion behavior of Al/Mg FSW joints to improve the service performance. However, there is little research on the corrosion behavior of Al/Mg FSW joints modified by interlayers. It is known that Zr has high thermal stability and can act as a barrier layer to mitigate the strong reaction at the Al/Mg interface. Moreover, Zr is an effective grain refinement element for both Al and Mg alloys, which can simultaneously improve their mechanical and corrosion properties. Considering the beneficial effects of Zr on Al and Mg alloys, the corrosion behavior of Al/Mg FSW joints may be improved by a Zr interlayer via microstructure optimization. To our knowledge, there are few studies on the preparation and characterization of Al/Mg FSW joints with a Zr interlayer. In this work, the microstructure and corrosion behavior of 6061 Al/AZ31 Mg FSW joints with a Zr interlayer was investigated. The results can reveal the effects of a Zr interlayer on the microstructure and corrosion behavior of Al/Mg FSW joints, which may provide design guidelines for improving joint corrosion properties by means of interlayers.

## 2. Materials and Methods

### 2.1. Materials Pretreatment

This study used 6061 Al and AZ31 Mg alloys with a size of 300 mm × 60 mm × 3 mm, provided by the Southwest Aluminum (Group) Co., Ltd (Chongqing, China). The corresponding chemical compositions are listed in Table 1. The 0.2 mm-thick Zr foil (99.9%), purchased from the General Research Institute for Nonferrous Metals of China, was selected as the interlayer. Before FSW, the base materials were mechanically polished using SiC emery papers with 500, 1000, 1500, 2000, and 3000 grits and ultrasonically cleaned in absolute ethanol.

### 2.2. FSW Process

**Figure 1:**
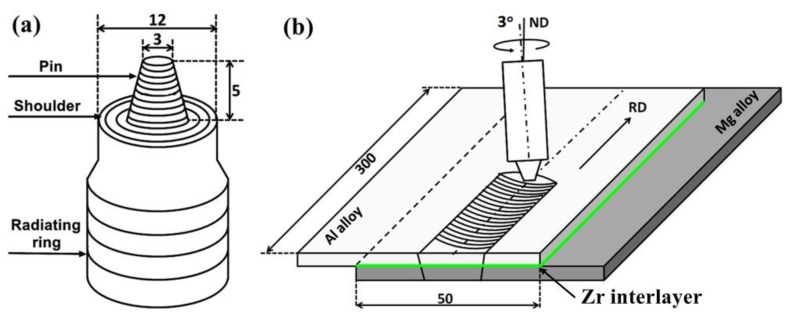
Schematic diagrams of (**a**) the friction stir welding (FSW) tool and (**b**) the welding process.

The 6061 Al alloy was lap joined to the AZ31 Mg alloy using a SCB-LM2217-2D-3T FSW machine (Beijing Sooncable Technology Development Co., Ltd., Beijing, China). The joint configuration with an overlap area of 300 mm × 50 mm was arranged as the AZ31 Mg alloy, the Zr interlayer, and the 6061 Al alloy from bottom to top, respectively. The size of the Zr interlayer was set as 300 mm × 50 mm × 0.2 mm. Figure 1 shows the schematic diagrams of the FSW tool and welding process. The FSW tool was composed of a dual-loop shoulder (12 mm in diameter) and a conical pin with threaded grooves (5 mm in length and 3 mm in root diameter). During FSW, the tool rotated anticlockwise and proceeded along the rolling direction (RD) with a tilt angle of 3° relative to the normal direction (ND). The shoulder plunge depth, rotation rate, and welding speed were 0.2 mm, 800 rpm, and 100 mm/min, respectively.

### 2.3. Microstructure Characterization

The joint appearances were captured by a Nikon D7200 digital camera (Bangkok, Thailand). The cross-sectional samples were cut into proper sizes by an electro-discharge machine and mechanically polished to mirror-like surfaces. The etching solutions were 20 g of NaOH with 100 mL of distilled water for Al alloy and 4% HNO_3_ ethanol for Mg alloy. The macroscopic morphologies were observed by the optical microscopy (OM, DSX-510, Olympus, Tokyo, Japan). The microstructures and chemical compositions were investigated via scanning electron microscopy (SEM, JSM 6480, JEOL, Tokyo, Japan) and energy dispersive X-ray spectrometer (EDS, Oxford Instruments, Abingdon, UK) in the secondary electron mode.

### 2.4. Electrochemical Measurements

The electrochemical measurements were conducted by an electrochemical workstation (CHI-660e, CH Instruments Inc., Shanghai, China) in 3.5% NaCl solution at 25 °C. A three-electrode system with the samples as the working electrode, the saturated calomel electrode (SCE) as the reference electrode, and the platinum sheet as the counter electrode was employed. The FSW joints exhibited characteristic microstructures with four typical regions: the base material (BM), the heat-affected zone (HAZ), the thermo-mechanically affected zone (TMAZ), and the stir zone (SZ) [17,18]. Three representative regions of BM, HAZ, and SZ of the FSW joints were tested with no particular attempt to discriminate the HAZ and TMAZ because of their indistinct boundary [19]. The testing areas were exposed with the other areas sealed by the chloroprene rubber. The open circuit potential (OCP) curves were recorded at a sample interval of 0.1 s for 30 min to achieve stabilization at the surface/solution interface. The potentiodynamic polarization curves were measured at a scan rate of 1 mV·s^−1^. The corrosion current density (*i*_corr_) was calculated in the Tafel linear region by the CHI-660e software at 60 mV relative to the corrosion potential (E_corr_). Three measurements from each group were taken to calculate the average. 

### 2.5. Immersion Tests

Immersion tests were performed in the 3.5% NaCl solution at 25 °C to investigate the changes in corrosion morphologies and corrosion products. After specific immersion times, the samples were taken out, rinsed with distilled water, and dried in air. The corrosion products were analyzed by X-ray diffractometer (XRD, D8 Advance, Bruker, Karlsruhe, Germany) using Cu k_α_ radiation in the 2θ range of 10–90° at a scan rate of 6°/min. The corroded surfaces were analyzed by SEM (Quanta 450 FEG, FEI, Hillsboro, OR, USA) and its EDS attachment. The weight losses of joints were measured with a scale (0.01 mg in accuracy) after removing the corrosion products using the 200 g·L^−1^ CrO_3_ solution.

## 3. Results and Discussion

### 3.1. Weld Appearances and Macrostructures

**Figure 2:**
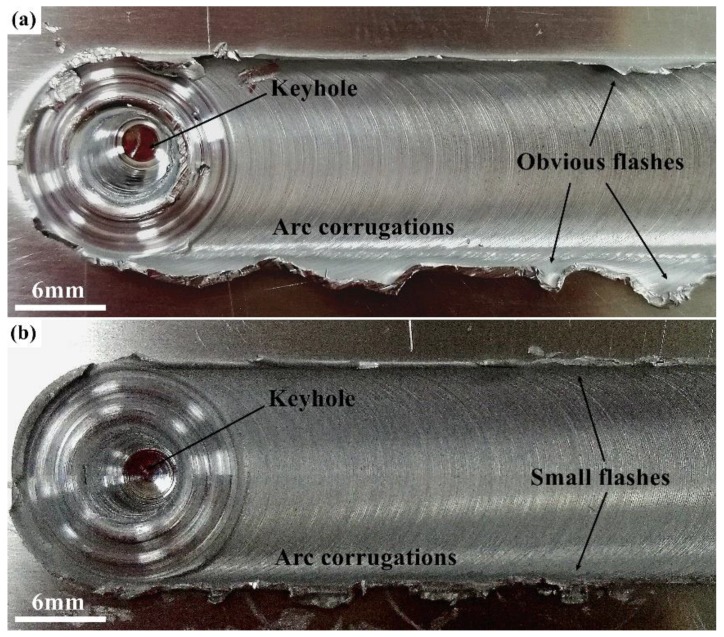
Surface appearances of (**a**) Al/Mg and (**b**) Al/Zr/Mg joints.

Figure 2 shows the surface appearances of the Al/Mg and Al/Zr/Mg joints. In Figure 2a, the surface of the Al/Mg joint is featured by obvious flashes on the weld edges. These flashes are produced in the excreting course of deformed materials from the joint to the surface [20,21]. The Al/Zr/Mg joint has a relatively smooth surface with small flashes, as shown in Figure 2b. Homogeneous arc corrugations are observed for both joints, which are formed by the frictional interaction between the rotating tool shoulder and the material surface. Typical keyholes are also generated at the end of the welds as the tool pin retracts from the joint.

**Figure 3:**
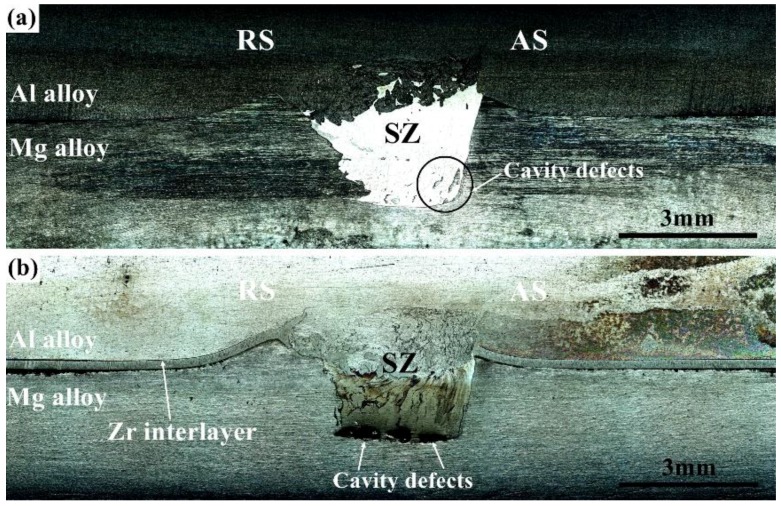
Cross-sectional profiles of the (**a**) Al/Mg and (**b**) Al/Zr/Mg joints.

Figure 3 presents the cross-sectional profiles of the Al/Mg and Al/Zr/Mg joints. As shown in Figure 3a, the Al/Mg joint was developed via the mutual movements of the base materials assisted by the tool pin, which formed a cone-shape SZ to interlock the Al and Mg alloys. For the Al/Zr/Mg joint, as shown in Figure 3b, the central part of the Zr interlayer was stirred into the SZ by the tool pin with the undamaged part being sandwiched at the Al/Mg interface. The SZ of both joints exhibited a non-symmetric structure with the advancing side (AS) showing a steeper edge than the retreating side (RS), which was due to the more severe material flows on the AS [22]. It is noted that some cavity defects were observed at the interface between the SZ and the Mg alloy for both joints, which may have been caused by the insufficient frictional heat and low deformability of the Mg alloy [5,23]. These cavities are harmful for the joint quality and will be reduced via optimization of the rotation rate and welding speed in the future work. 

### 3.2. Microstructures

**Figure 4:**
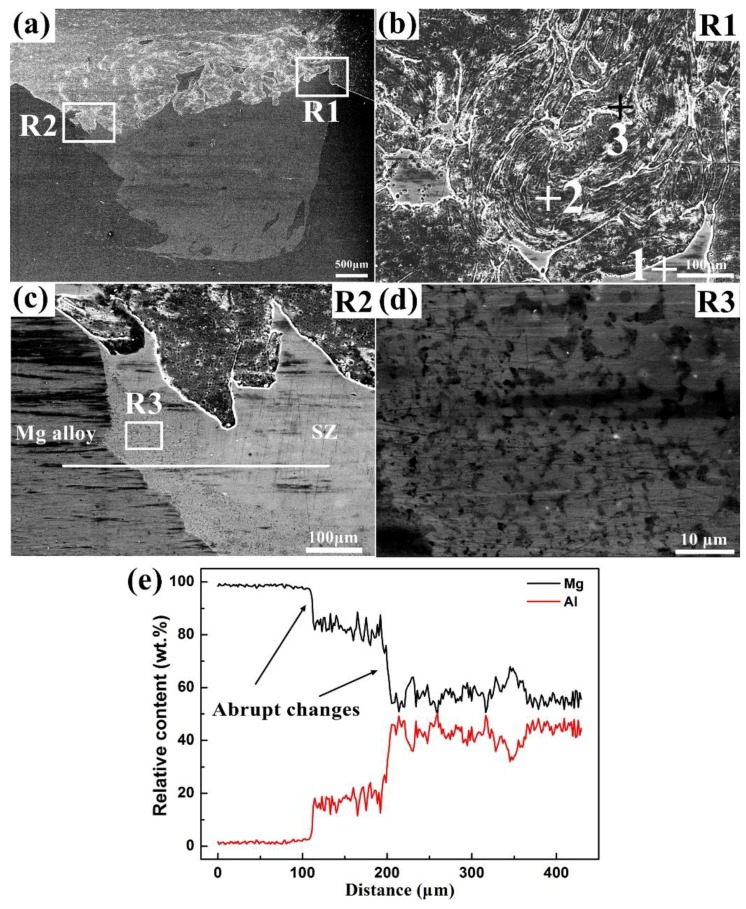
Representative microstructures of the Al/Mg joint: (**a**) overview of the SZ, (**b**–**d**) magnified views of the R1, R2, and R3, respectively, and (**e**) EDS line-scan results of the R2.

Figure 4 depicts the representative microstructures and EDS line-scan results of the Al/Mg joint. In Figure 4a, the SZ of the Al/Mg joint is constituted by a chaotic morphology on the top and a uniform morphology on the bottom. The magnified view of the R1 region in Figure 4b reveals the existence of vortex-like Al–Mg IMCs in the SZ. The elemental compositions at points 1–3 in Figure 4b are listed in Table 2. The gray zone, dark zone, and white zone, marked as points 1, 2, and 3 are primarily Al_3_Mg_2_, an Al matrix with little Al_2_O_3_, and Al_12_Mg_17_ with a lot of Al_2_O_3_ and MgO, respectively. Similar results can also be found in other studies of Al/Mg FSW joints [24,25]. The detection of metallic oxides may have originated from two sources [26]: (1) the thermal interaction between oxygen and the base materials and (2) the remaining oxide layers on the base materials, which were mixed into the SZ. The magnified view of the R2 region in Figure 4c exhibits an intercalated microstructure with a wide transition zone at the Mg alloy/SZ interface. The magnified view of the R3 region in Figure 4d indicates that there existed a lot of irregular Al–Mg IMCs in the transition zone. The corresponding EDS line-scan results in Figure 4e show the formation of abrupt changes in elemental contents of Al and Mg between adjacent plateaus, suggesting the presence of stable Al–Mg IMCs. The Al–Mg IMCs were formed by the constitutional liquation phenomenon via the eutectic reactions of L = Al + Al_3_Mg_2_ at 450 °C and L = Mg + Al_12_Mg_17_ at 437 °C, respectively [27,28,29].

**Figure 5:**
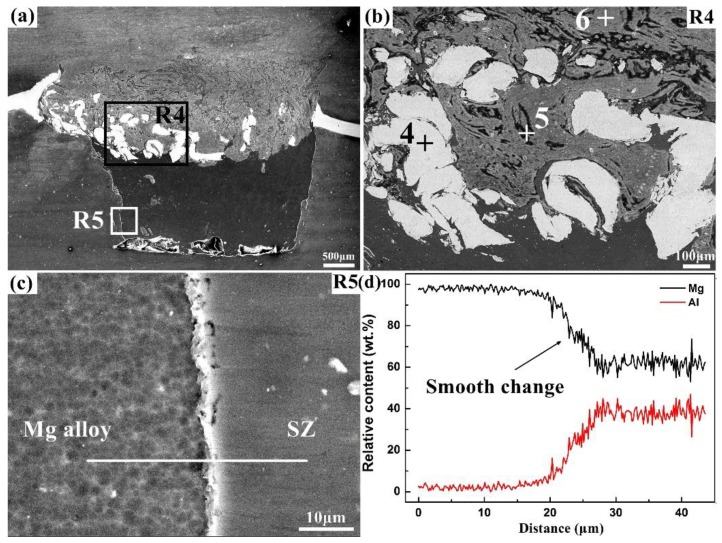
Representative microstructures of the Al/Zr/Mg joint: (**a**) overview of the stir zone (SZ), (**b**,**c**) magnified views of the R4 and R5, respectively, and (**d**) EDS line-scan results of the R4.

Figure 5 demonstrates the representative microstructures and EDS line-scan results of the Al/Zr/Mg joint. As shown in Figure 5a, the smashed Zr interlayer distributed in the SZ was intermixed with the base materials, while the undamaged part remained stable at the Al/Mg interface. The magnified view of the R4 region in Figure 5b displayed three different morphologies of white plates, dark strips, and gray matrix in the SZ. The elemental compositions at points 4–6 in Figure 5b are summarized in Table 3. The white plates, dark strips, and gray matrix marked as points 4, 5, and 6 are Zr with little ZrO_2_, Al matrix with a lot of Al_2_O_3_ and MgO, Al matrix with little Al_2_O_3_, respectively. The magnified view of the R5 region in Figure 5c indicates the existence of a thin transition zone at the Mg alloy/SZ interface. The corresponding EDS line-scan results in Figure 5d exhibit a smooth transition in Al and Mg content with no abrupt changes from the Mg alloy to the SZ.

In comparison with the Al/Mg joint (see Figure 4 and Table 2), the Al/Zr/Mg joint revealed more favorable elemental distributions with reduced Al–Mg IMCs (see Figure 5 and Table 3). This microstructure improvement may be explained by the synergetic effects of the Zr interlayer: (1) The smashed Zr intermixed in the SZ exhibited a chemical modification effect to hinder the strong reaction between the base materials, as evidenced by the much lower Mg content in the R4 region than that in the R1 region; and (2) the undamaged Zr at the Al/Mg interface segregated the contact of the base materials, which showed a thermal barrier effect in mitigating the mutual diffusion of Al and Mg. 

### 3.3. Corrosion Behavior

**Figure 6:**
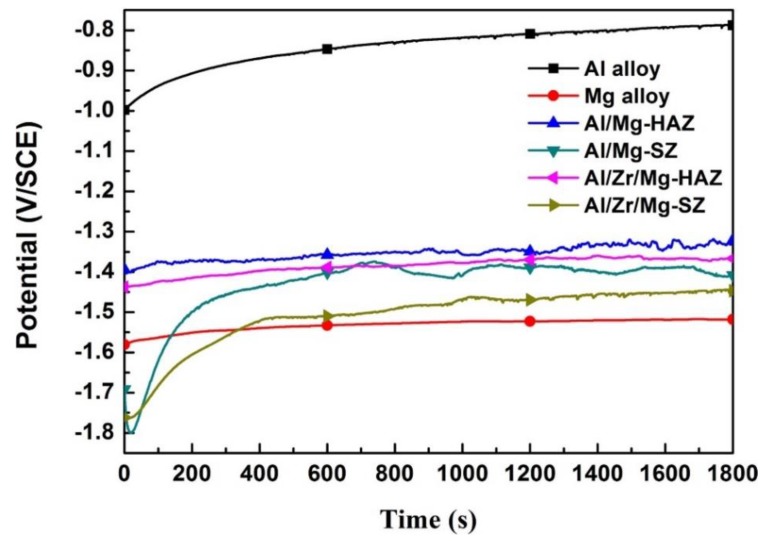
The open circuit potential (OCP) curves for the representative regions of the Al/Mg and Al/Zr/Mg joints in the 3.5% NaCl solution.

Figure 6 presents the OCP curves for the representative regions of the Al/Mg and Al/Zr/Mg joints in the 3.5% NaCl solution. The OCP curves of the experimental samples exhibited a rising trend at the initial stage and then became steady at the final stage, indicating that the chemical status on the surface gradually became stable with the increasing immersion time. The highest and lowest OCP values can be found for the Al alloy and Mg alloy, respectively. The OCP values of the Al/Mg-HAZ, Al/Mg-SZ, Al/Zr/Mg-HAZ, and Al/Zr/Mg-SZ samples were within the range of the base materials. This variation in OCP values may be related to the passivation layers on the surface. The compact Al_2_O_3_ layer on the Al alloy surface had some anti-corrosion ability, while the porous MgO layer on the Mg alloy surface was easily dissolved by the corrosive media. The passivation layers of the representative regions (HAZ and SZ) were composed of metallic oxide mixtures, such as Al_2_O_3_, MgO, and ZrO_2_, which presented a mediate passivation effect. Moreover, there appeared to be some fluctuations in the OCP curves, which were caused by the dissolution–regeneration process of the passivation layers [30].

**Figure 7:**
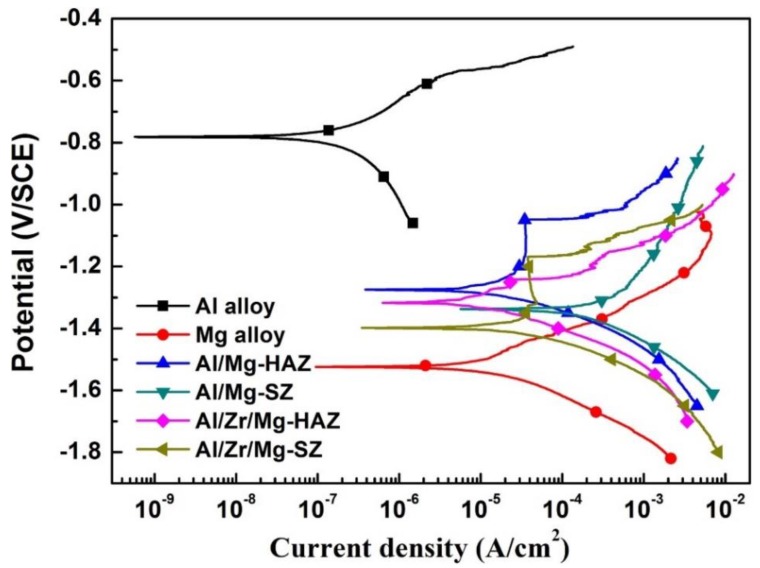
Potentiodynamic polarization curves for the representative regions of the Al/Mg and Al/Zr/Mg joints in the 3.5% NaCl solution.

Figure 7 displays the potentiodynamic polarization curves for the representative regions of the Al/Mg and Al/Zr/Mg joints in the 3.5% NaCl solution. The corresponding electrochemical parameters of E_corr_ and *i*_corr_ are listed in Table 4. Usually, the E_corr_ is a thermodynamic indicator to estimate the corrosion probability, and a larger E_corr_ represents a higher surface stability. On the other hand, the *i*_corr_ is a kinetic indicator to evaluate the corrosion degree once corrosion occurs, and a smaller *i*_corr_ indicates a lower corrosion rate [31,32]. The Al alloy had a higher E_corr_ and a lower *i*_corr_ compared with the Mg alloy, suggesting that superior corrosion resistance was found for the Al alloy. The E_corr_ of the Al/Mg-HAZ, Al/Mg-SZ, Al/Zr/Mg-HAZ, and Al/Zr/Mg-SZ samples were between those of the Al alloy and the Mg alloy, which were the mixed potentials of the base materials. The *i*_corr_ increased in the order of Al alloy < Mg alloy < Al/Zr/Mg-HAZ < Al/Mg-HAZ < Al/Zr/Mg-SZ < Al/Mg-SZ samples, implying the same change trend in the corrosion rate. For both Al/Mg and Al/Zr/Mg joints, the corrosion rate of the representative regions decreased in the order of SZ > HAZ > base materials. This phenomenon may have been owing to the following two reasons: (1) The SZ and HAZ, incorporating Al and Mg dissimilar alloys, experienced galvanic corrosion at the Al/Mg interface because of the potential differences, which induced faster corrosion than the base materials; and (2) different from the HAZ, the SZ possessed a more complex microstructure with heterogeneous phases of Al–Mg IMCs, metals, and metallic oxides, which accelerated the corrosion rate via building more local corrosion cells. In addition, the corrosion behavior of the Al/Mg joint was improved by the Zr interlayer, as could be seen from the smaller *i*_corr_ at the HAZ and SZ for the Al/Zr/Mg joint. The Zr interlayer reduced the formation of Al–Mg IMCs in the SZ and segregates the contact of the base materials at the interface (see Figure 5 and Table 3), which mitigated the galvanic corrosion effect between the Al and Mg alloys.

**Figure 8:**
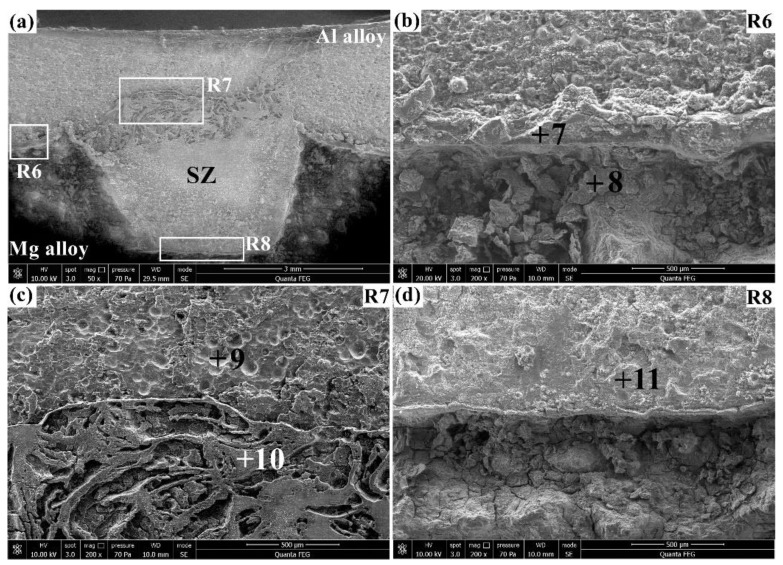
Corrosion morphologies of the Al/Mg joint in the 3.5% NaCl solution for 4 h: (**a**) overview and (**b**–**d**) magnified views of the R6, R7, and R8, respectively.

Figure 8 shows the corrosion morphologies of the Al/Mg joint after immersion in the 3.5% NaCl solution for 4 h. The corresponding elemental compositions of points 7–11 are summarized in Table 5. It must be pointed out that SEM-EDS is a semi-quantitative measuring technique, and it may cause deviations in quantifying the minor elements. In order to reduce this inaccuracy, the contents of minor elements (Cl, Si, and Zn) are added up and listed as the “others”. The corrosion behavior of the joints were analyzed by comparing the contents of the major elements (Al, Mg, O, and Zr). This similar analysis method has also been reported in [29], where the phase constitutions of Al/Mg dissimilar FSW joints were investigated via the major elements (Al and Mg) with the minor elements (Fe, Mn, Si, Zn, etc.) added as a whole. As shown Figure 8a, the Al/Mg joint was corroded to form diverse corrosion morphologies at different regions. The magnified view of the R6 region in Figure 8b indicates that the Al alloy with a flat surface had a higher corrosion resistance than the Mg alloy with a rough surface, which was due to the galvanic corrosion between active Mg and noble Al [15]. The corrosion products at the Al/Mg interface (point 7) were mainly composed of O and Mg with a small amount of Al, implying the formation of Mg oxides. The corroded surface of the Mg alloy (point 8) was filled with blocky corrosion products, whose composition had slightly lower Al and O with higher Mg compared with the Al/Mg interface (point 7). The magnified view of the R7 region in Figure 8c is featured with two distinct corrosion forms: the uniform corrosion at the Al alloy (point 9) and the discrete corrosion on the top of the SZ (point 10). The granular corrosion morphology at point 9 contained 60.01 Al, 4.85 Mg, 29.46 O, and 5.68 minor elements (in wt.%) due to the corrosion of the Al alloy, while the dendritic corrosion morphology at point 10 included 49.85 Al, 31.27 Mg, 17.28 O, and 1.60 minor elements (in wt.%), owing to the combined corrosion of the base materials and Al–Mg IMCs. The magnified view of the R8 region in Figure 8d demonstrates that the SZ on the bottom had a compact corrosion morphology, whereas the adjacent Mg alloy was badly corroded to form many cracks and some holes. High O and Mg with trace Al were detected on the bottom of the SZ (point 11), suggesting the existence of Mg oxides formed by the corrosion of the Mg alloy.

**Figure 9:**
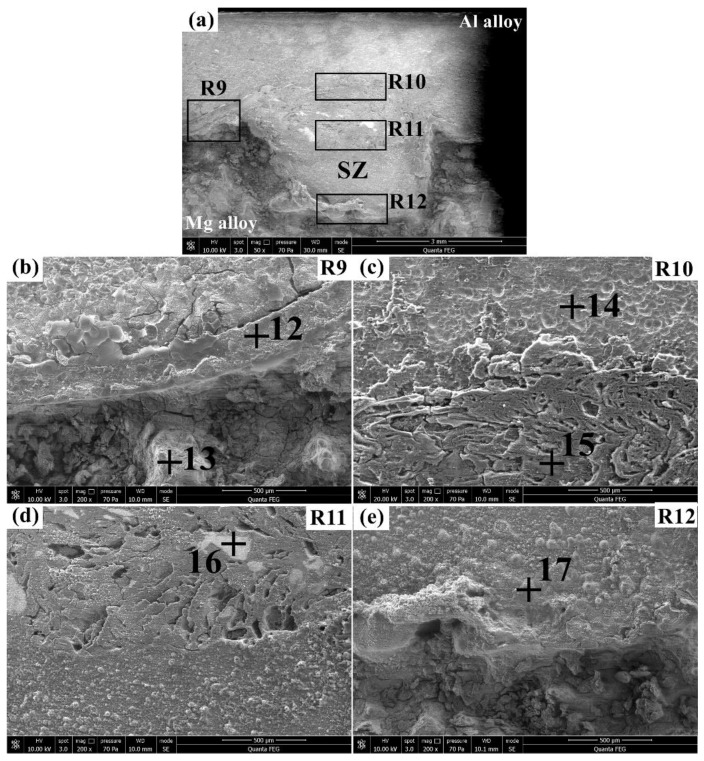
Corrosion morphologies of the Al/Zr/Mg joint in the 3.5% NaCl solution for 4 h: (**a**) overview and (**b**–**e**) magnified views of the R9, R10, R11, and R12, respectively.

The corrosion morphologies of the Al/Zr/Mg joint after immersion in the 3.5% NaCl solution for 4 h are exhibited in Figure 9. Table 6 lists the corresponding elemental compositions of points 12–17. In Figure 9a, an inhomogeneous corrosion morphology can be observed for the Al/Zr/Mg joint with the appearance of some Zr pieces. The magnified view of the R9 region in Figure 9b shows a sandwiched microstructure with the Al and Mg alloys being separated by the Zr interlayer, which blocks the contact of the base materials and retards the galvanic corrosion at the Al/Mg interface to some degree. The corroded surface of the Zr interlayer (point 12) was characterized by high O and Zr with some Al and Mg, indicating that the main corrosion products were Zr oxides. The blocky corrosion products of the Mg alloy (point 13) have higher Mg and comparable Al and O compared with point 8 (see Figure 8b), implying that the corrosion of the Mg alloy was slightly mitigated. The magnified view of the R10 region in Figure 9c presents a corrosion morphology similar to that of the R7 region of the Al/Mg joint. The corrosion products of the Al alloy at point 14 were composed of 81.37 Al, 1.89 Mg, 14.53 O, and 2.21 minor elements (in wt.%), indicating that a lower corrosion degree than point 9 (see Figure 8c) was obtained. The corroded surface on the top of the SZ (point 15) showed a relatively uniform and continuous corrosion morphology with comparable compositions of 47.39 Al, 29.43 Mg, 22.29 O, and 0.89 minor elements (in wt.%) to those of point 10 (see Figure 8c). The magnified view of the R11 region in Figure 9d shows that many irregular Zr pieces were embedded in the middle of the SZ, whose composition primarily consisted of Zr and O with little Al and Mg (point 16). The magnified view of the R12 region in Figure 9e exhibits a corrosion morphology similar to that of the R8 region of the Al/Mg joint. The corrosion degree of the SZ on the bottom (Point 17) was comparable to that of point 11 (see Figure 8d), as can be seen by their similar compositions. 

**Figure 10:**
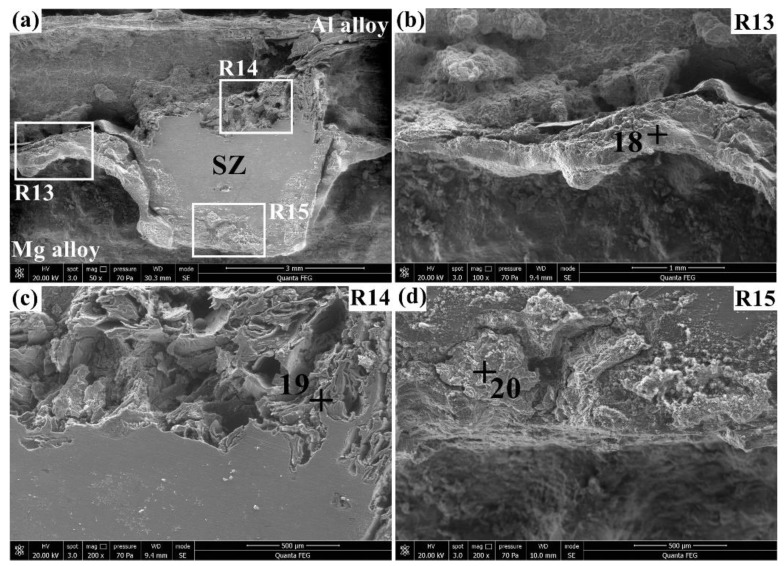
Corrosion morphologies of the Al/Mg joint in the 3.5% NaCl solution for 60 h: (**a**) overview and (**b**–**d**) magnified views of the R13, R14, and R15, respectively.

Figure 10 displays the corrosion morphologies of the Al/Mg joint after immersion in the 3.5% NaCl solution for 60 h. The corresponding elemental compositions of points 18–20 are listed in Table 7. As shown in Figure 10a, the corrosion degree of the Al/Mg joint was aggravated as the immersion time increased to 60 h. The magnified view of the R13 region in Figure 10b shows that more severe corrosion was found for the Al and Mg alloys compared with the Al/Mg interface. The corroded surface at the Al/Mg interface (point 18) had a similar composition with that of point 7 (see Figure 8b), indicating that no further corrosion was obviously observed at the Al/Mg interface as immersion time increased. The magnified view of the R14 region in Figure 10c exhibits a discrete corrosion morphology on the top of the SZ, which produced a discontinuous surface with many irregular grooves. The dendritic corrosion morphology at point 19 had a heavier corrosion degree than point 10 (see Figure 8c), as evidenced by its lower Al, Mg, and higher O. The magnified view of the R15 region in Figure 10d demonstrates that the Mg alloy corroded faster than the SZ on the bottom to form an uneven surface with a large height difference. Relatively higher Al and lower Mg were detected on the bottom of the SZ (point 20) than at point 11 (see Figure 8d), which resulted from the further corrosion of the Mg alloy. 

**Figure 11:**
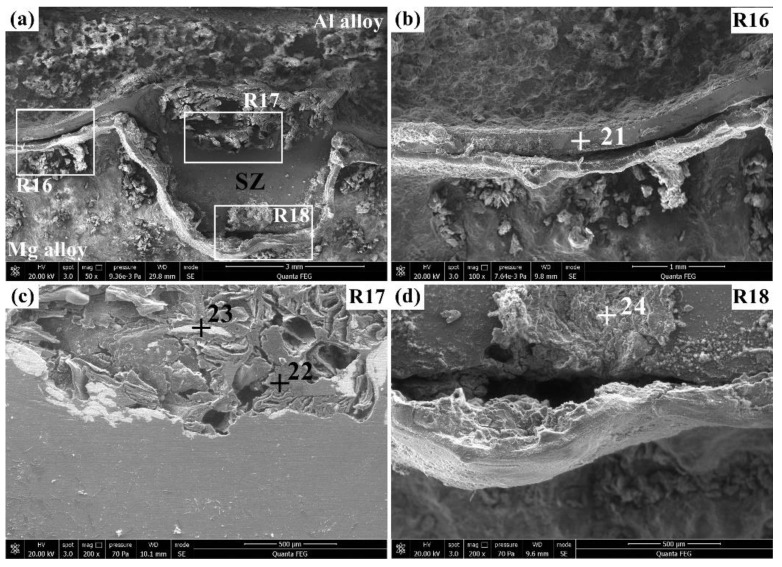
Corrosion morphologies of the Al/Zr/Mg joint in the 3.5% NaCl solution for 60 h: (**a**) overview and (**b**–**d**) magnified views of the R16, R17, and R18, respectively.

The corrosion morphologies of the Al/Zr/Mg joint after immersion in the 3.5% NaCl solution for 60 h are presented in Figure 11. Table 8 summarizes the corresponding elemental compositions of points 21–24. As shown in Figure 11a, relatively mild corrosion with a flatter corroded morphology was found in the Al/Zr/Mg joint compared with that of the Al/Mg joint after 60 h of immersion time (see Figure 10a). The magnified view of the R16 region in Figure 11b shows that the Zr interlayer remained stable at the Al/Mg interface to balance the corrosion of the base materials, as can be seen from the inconspicuous height difference between the Al and Mg alloys. The corroded surface of the Zr interlayer (point 21) had a higher Al and Mg and a much lower Zr than that at point 12 (see Figure 9b), implying the further corrosion of the Zr interlayer. The magnified view of the R17 region in Figure 11c exhibits that the SZ on the top was corroded to form some small pits with the existence of dendritic corrosion products and Zr pieces. The dendritic corrosion products at point 22 were characterized by a much lower Al, higher O, and similar Mg compared with that of point 19 (see Figure 10c), suggesting that the Al alloy was further corroded to form metallic oxides on the top of the SZ. The Zr pieces at point 23 had similar compositions with those at point 16 (see Figure 9d), indicating that no further corrosion was observed for the Zr pieces. The magnified view of the R18 region in Figure 11d demonstrates that there was a transverse crevice on the bottom of the SZ, which may have provided passages for corrosive media to further corrosion. The corrosion products on the bottom of the SZ (point 24) had comparable compositions with those at point 20 (see Figure 10d), suggesting a similar corrosion degree for both sites. 

**Figure 12:**
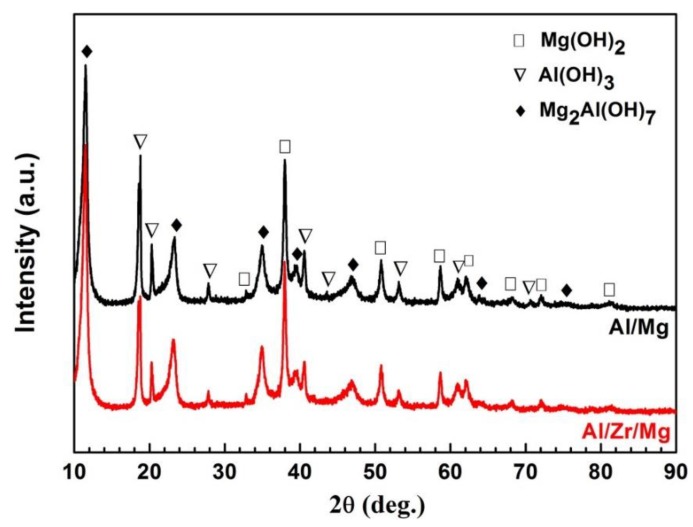
XRD patterns for the corrosion products of the Al/Mg and Al/Zr/Mg joints.

Figure 12 presents the XRD patterns for the corrosion products of the Al/Mg and Al/Zr/Mg joints. No obvious differences in XRD patterns were observed between the Al/Mg and Al/Zr/Mg joints, suggesting that the phase constitutions of the corrosion products for both joints were similar. The diffraction peaks of the corrosion products were identified as Mg(OH)_2_, Al(OH)_3_, and Mg_2_Al(OH)_7_ phases by MDI Jade 6.0 software, which correspond to the standard PDF #83-0114, #20-0011, and #35-1275, respectively. The corrosion of Al and Mg alloys usually produces metallic oxides, which are easily transformed to metallic hydroxides under humid environment [33,34]. Furthermore, no Zr-containing phase was detected because of its low content. The detection of metallic hydroxides by the XRD patterns was inconsistent with the EDS results, which was attributed to the inability of EDS to detect H.

**Figure 13:**
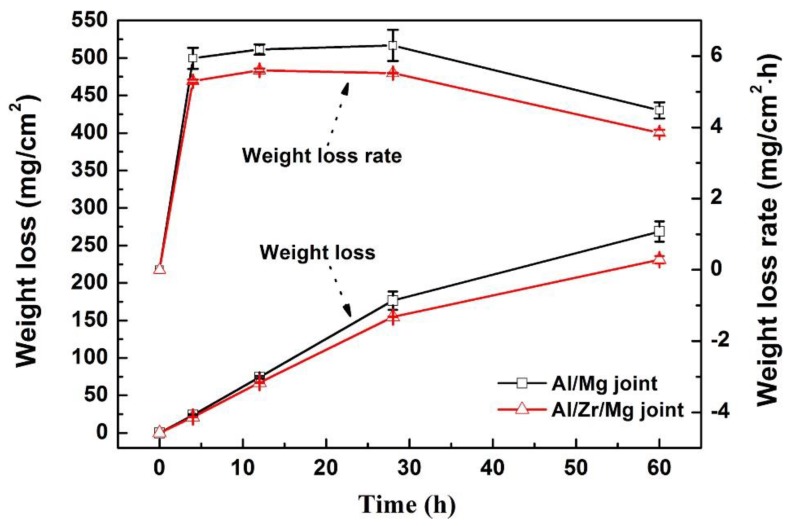
Weight losses and corresponding change rates of the Al/Mg and Al/Zr/Mg joints during the immersion tests in the 3.5% NaCl solution for 60 h.

Figure 13 illustrates the weight losses and corresponding change rates of the Al/Mg and Al/Zr/Mg joints during the immersion tests in the 3.5% NaCl solution for 60 h. The weight loss of the Al/Mg joint linearly increased to 176.42 ± 12.22 mg/cm^2^ in the initial 28 h and slowly increased to 268.72 ± 13.44 mg/cm^2^ in the last 32 h. The weight loss of the Al/Zr/Mg joint reached 231.08 ± 4.81 mg/cm^2^ during the whole 60 h and exhibited a 14% reduction compared with that of the Al/Mg joint, which evidenced that the corrosion resistance was enhanced by the Zr interlayer. Moreover, the weight loss rate of the Al/Mg joint slowly increased to 6.30 ± 0.44 mg/cm^2^/h with increasing immersion time to 28 h and then decreased to 4.48 ± 0.22 mg/cm^2^/h at the end of 60 h. A similar change trend in weight loss rate was found for the Al/Zr/Mg joint except for the slightly lower value at each immersion time. The slowdown in weight loss rate may be attributed to the gradual deposition of corrosion products on the surface, which can act as a protective layer to suppress further corrosion [35]. 

## 4. Conclusions

In this work, the microstructure and corrosion behavior of friction stir-welded 6061 Al/AZ31 Mg joints with Zr interlayer were studied. The following conclusions were drawn:

(1) Dissimilar alloys of 6061 Al and AZ31 Mg were successfully joined by FSW with a Zr interlayer. The central part of the Zr interlayer was smashed and intermixed with the base materials in the SZ, and the undamaged part remained stable at the Al/Mg interface. The Zr interlayer improved the elemental distributions to exhibit reduced Al–Mg IMCs in the SZ and a thinner transition zone at the interface, which was attributed to its chemical modification and thermal barrier effects.
(2) A differentiated corrosion behavior was found for the representative regions of the joints, whose corrosion rate increased in the order of Al alloy < Mg alloy < Al/Zr/Mg-HAZ < Al/Mg-HAZ < Al/Zr/Mg-SZ < Al/Mg-SZ. The SZ with heterogeneous phases of Al–Mg IMCs, metals, and metallic oxides showed a larger corrosion rate than the HAZ for each joint, which was owing to its more local corrosion cells than the HAZ.
(3) The Zr interlayer enhanced the corrosion resistance of the Al/Mg joint to present a 14% reduction in weight loss for the Al/Zr/Mg joint after immersion tests in the 3.5% NaCl solution, which was ascribed to its beneficial effect in mitigating the galvanic corrosion between the base materials. The corrosion products of the Al/Mg and Al/Zr/Mg joints were mainly composed of Mg(OH)_2_, Al(OH)_3_, and Mg_2_Al(OH)_7_ phases.

Our future work will investigate the relationship between the joint microstructure, corrosion behavior, and mechanical properties under various combinations of FSW parameters and Zr interlayer thicknesses to prepare high-quality Al/Mg dissimilar joints.

## Figures and Tables

**Table 1:** Chemical compositions (in wt.%) of the 6061 Al and AZ31 Mg alloys.

Materials	Al	Mg	Si	Fe	Cu	Cr	Mn	Zn	Ti	Ni
6061	Bal.	1.00	0.73	0.30	0.28	0.16	0.05	0.05	0.01	--
AZ31	3.20	Bal.	0.05	0.0022	0.0018	--	0.35	0.82	--	0.00016

**Table 2:** Elemental compositions (in wt.%) at points 1–3 in Figure 4b.

Points	Al	Mg	O	Total	Inference Phases
1	61.01	36.18	2.81	100.00	Al_3_Mg_2_
2	90.44	0.98	8.58	100.00	Al matrix + Al_2_O_3_
3	44.38	19.42	36.20	100.00	Al_12_Mg_17_ + Al_2_O_3_ + MgO

**Table 3:** Elemental compositions (in wt.%) at points 4–6 in Figure 5b.

Points	Al	Mg	O	Zr	Total	Inference Phases
4	--	--	7.38	92.62	100.00	Zr + ZrO_2_
5	64.16	8.03	27.81	--	100.00	Al matrix + Al_2_O_3_ + MgO
6	92.41	1.05	6.54	--	100.00	Al matrix + Al_2_O_3_

**Table 4:** Electrochemical parameters fitted from the potentiodynamic polarization curves.

Samples	Al Alloy	Mg Alloy	Al/Mg-HAZ	Al/Mg-SZ	Al/Zr/Mg-HAZ	Al/Zr/Mg-SZ
E_corr_ (V/SCE)	−0.77 ± 0.01	−1.51 ± 0.02	−1.27 ± 0.03	−1.36 ± 0.02	−1.31 ± 0.01	−1.43 ± 0.03
*i*_corr_ (μA/cm^2^)	0.23 ± 0.03	12.41 ± 1.66	29.30 ± 2.95	320.50 ± 22.10	13.92 ± 1.50	60.18 ± 2.95

**Table 5:** Elemental compositions (in wt.%) of points 7–11 in Figure 8b–d.

Points	Al	Mg	O	Others	Total
7	8.54	30.31	56.91	4.24	100.00
8	2.81	34.80	52.45	9.94	100.00
9	60.01	4.85	29.46	5.68	100.00
10	49.85	31.27	17.28	1.60	100.00
11	0.73	43.71	53.78	1.78	100.00

**Table 6:** Elemental compositions (in wt.%) of points 12–17 in Figure 9b–e.

Points	Al	Mg	O	Zr	Others	Total
12	3.35	10.32	50.70	35.03	0.60	100.00
13	2.62	40.64	54.69	--	2.05	100.00
14	81.37	1.89	14.53	--	2.21	100.00
15	47.39	29.43	22.29	--	0.89	100.00
16	3.19	3.91	34.72	57.16	1.02	100.00
17	2.56	44.31	53.13	--	--	100.00

**Table 7:** Elemental compositions (in wt.%) of points 18–20 in Figure 10b–d.

Points	Al	Mg	O	Others	Total
18	5.29	39.42	55.29	--	100.00
19	40.14	27.20	29.22	3.44	100.00
20	9.62	33.13	55.63	1.62	100.00

**Table 8:** Elemental compositions (in wt.%) of points 21–24 in Figure 11b–d.

Points	Al	Mg	O	Zr	Others	Total
21	20.65	18.76	58.12	1.53	0.94	100.00
22	16.98	25.13	53.94	1.49	2.46	100.00
23	2.30	3.76	34.45	59.49	--	100.00
24	8.47	30.96	58.08	--	2.49	100.00
